# On Poisons in Relation to Medical Jurisprudence

**Published:** 1876-02

**Authors:** 


*On Poisons in Relation to Medical Jurisprudence and Medicine.* By Alfred Swaine Taylor, M. D., F. R. S. Third American from the third and thoroughly revised English edition. With 104 illustrations. Philadelphia: Henry C. Lea, 1876. Buffalo: T. Butler & Son.

The previous editions of this work, owing to the rapid advances constantly made in chemical and physical science, have fallen behind the times; and in order to meet the demand for a manual in harmony with the present condition of science, the author has thoroughly revised, and, in some instances, entirely rewritten portions of the present edition.

It is not presented to the profession as a history of poisons or poisoning, but as a simple manual for the use of students of law or medicine desiring to refer to such a work.

The author is a recognized authority on the subject of medical jurisprudenca, and is widely quoted by other writers. The two former editions have been so favorably received, and at the time of their publication were so well in accord with what was best known in the department of which they treated, that we need only call attention to the publication of the present revised edition.

